# Pueraria lobata Potentially Treating Prostate Cancer on Single-Cell Level by Network Pharmacology and AutoDock: Clinical Findings and Drug Targets

**DOI:** 10.1155/2022/3758219

**Published:** 2022-11-21

**Authors:** Yongfeng Mo, Manying Chen, Honyu Qin, Huaying Liu, Yu Ye

**Affiliations:** Department of Emergency, The Second Affiliated Hospital of Guangxi Medical University, Nanning, Guangxi 53000, China

## Abstract

**Background:**

Prostate cancer (PCa) is one of the common malignant tumors of the urological system, and metastasis often occurs in advanced stages. Chemotherapy is an effective treatment for advanced PCa but has limitations in terms of efficacy, side effects, multidrug resistance, and high treatment costs. Therefore, new treatment modalities for PCa need to be explored and improved.

**Methods:**

R language and GEO database were used to obtain differentially expressed genes for PCa single-cell sequencing. TCMSP, STITCH, SwissTargetPrediction, and PubChem databases were used to obtain the active ingredients and targets of *Pueraria lobata* (PL). Next, Cytoscape software was used to draw the interactive network diagram of “drug–active component–target pathway.” Based on the STRING database, the protein–protein interaction network was constructed. Gene Ontology and the Kyoto Encyclopedia of Genes and Genomes were applied for the genes. Molecular docking was used to visualize the drug–target interaction via AutoDock Vina and PyMOL. Finally, prognosis-related genes were found by survival analysis, and Protein Atlas was used for validation.

**Results:**

Four active components and 31 target genes were obtained through the regulatory network of PL. Functional enrichment analysis showed that PL played a pharmacological role in the treatment of PCa by regulating the metabolic processes of reactive oxygen species, response to steroid hormones, and oxidative stress as well as IL-17 signaling pathway, PCa, and estrogen signaling pathway. Single-cell data showed that *AR*, *MIF*, *HSP90B1*, and *MAOA* genes were highly expressed, and molecular docking analysis showed that representative components had a strong affinity with receptor proteins. Survival analysis found that *APOE*, *CA2*, *IGFBP3*, *MIF*, *F10*, and *NR3C1* could predict progression-free survival (PFS), and some of them could be validated in PCa.

**Conclusion:**

In this paper, a drug–active ingredient–target pathway network of PL at the single-cell level of PCa was constructed, and the findings revealed that it acted on genes such as *AR*, *MIF*, *HSP90B1*, and *MAOA* to regulate several biological processes and related signaling pathways to interfere with the occurrence and development of PCa. *APOE*, *CA2*, *IGFBP3*, *MIF*, *F10*, and *NR3C1* were also important as target genes in predicting PFS.

## 1. Introduction

Prostate cancer (PCa) is one of the common malignant tumors of the urinary system, and nearly 1.3 million new cases of PCa and 359,000 related deaths were estimated worldwide in 2018, ranking second and fifth in incidence and mortality rates of cancer in males, respectively [1]. In recent years, the incidence of PCa has been increasing in some Asian countries, especially in Northeast Asia. Although the diagnostic techniques for PCa are advancing, effective treatments are still lacking [2]. The conventional treatment for PCa mainly includes surgery, chemotherapy, and radiotherapy, which has limitations in terms of efficacy, side effects, multidrug resistance, and high treatment costs [3]. Therefore, exploring and improving the treatment of PCa is necessary.

Traditional Chinese Medicine (TCM) is a treasure house of potential drugs. Studies have shown that TCM has its unique advantages on the complex pathogenic mechanism of cancer. Therefore, the study of the mechanism of TCM may contribute to the development of a new combination of Chinese and Western medicine therapy [4]. Network pharmacology studies the interaction between biological systems, drugs, and diseases at the protein molecular and gene levels according to the target molecules, biological functions, and bioactive compounds and generates a complex interaction network, which conforms to the natural characteristics of TCM and can systematically clarify the mechanism of action of TCM at the molecular level [5]. *Pueraria lobata* (PL) is one of the TCM. Liu et al. showed that extracts of PL could induce apoptosis in PCa cells by upregulating the expression of *RASD1* and *Bax* [6]. In addition, PL extract inhibits cell proliferation by inhibiting the PI3K/AKT pathway and downregulating the expression of cyclin D1, *AKT*, and *CDK4* to induce cell cycle arrest in the G1 phase [7]. Studies have shown that a variety of active components of PL can play an antitumor role in cancer cell proliferation, cell cycle regulation, cell apoptosis, tumor angiogenesis, and metastasis [8, 9]. However, clear studies on the molecular mechanism of PL in relation to PCa are lacking.

Single-cell sequencing technology, which sequences the whole genome, transcriptome, and epigenome of individual cells, is significant in studying the differences and evolutionary relationships of the cell, revealing the complex heterogeneous mechanisms involved in disease onset and progression, and improving disease diagnosis, prognosis prediction, and monitoring of drug treatment effects [10, 11]. This paper is aimed at constructing a drug component–target pathway network by using network pharmacology combined with single-cell sequencing to explore the potential therapeutic effects of PL in PCa and its effect on prognosis. We present the following case in accordance with the CARE reporting checklist.

## 2. Methods

### 2.1. Differentially Expressed Genes Related to PCa

Thirteen cases of PCa single-cell RNA sequencing data in GSE141445 were obtained from the GEO database (https://www.ncbi.nlm.nih.gov/geo/) for inclusion in this paper. R language was used to define cell subsets according to marker genes, and cancer cell subsets were defined by copy number variation (CNV). Differentially expressed genes (DEGs) were obtained by FindAllMarkers function. DEG screening conditions were as follows: differential expression change fold change (FC) of mRNA, ∣logFC | >1, and adj. *P* < 0.05.

### 2.2. Screening of Active Components and Targets

The active ingredients and corresponding targets of PL were obtained using the TCMSP database (https://old.tcmsp-e.com/tcmsp.php). The screening conditions of active ingredients were as follows: oral bioavailability > 0.30 and drug‐likeness > 0.18. STITCH (http://stitch.embl.de/), SwissTargetPrediction (http://www.swisstargetprediction.ch/), and PubChem databases (https://pubchem.ncbi.nlm.nih.gov/) were used for complete PL composition target. The UniProt database (https://www.uniprot.org/) was used to transform IDs, and the final drug target was obtained after merging and deleting duplicates.

### 2.3. Construction of Drug Component–Target Gene Pathway Network and Topology Analysis

The obtained DEGs were intersected with the drug target to determine the target gene corresponding to the active ingredient, and the corresponding Kyoto Encyclopedia of Genes and Genomes (KEGG) pathway was further obtained through the target gene. On this basis, the drug component–target gene pathway network was constructed and visualized through Cytoscape. Topological parameters of the network are obtained through “CytoNCA” plugin of the Cytoscape software.

### 2.4. Functional Enrichment Analysis and Construction of Protein Interaction Networks

Gene Ontology (GO) and KEGG analyses were performed using clusterProfile, enrichplot, and ggplot packages in R language with PvalueCutoff = 0.05 and qvalueCutoff = 0.05 as the screening conditions. The results were presented as bubble plots. Protein interactions were analyzed by the STRING database (https://string-db.org/) for target genes, medium confidence > 0.4, and the results were visualized by Cytoscape.

### 2.5. Expression Levels of Target Genes in Single Cells and Molecular Docking

The coordinate mapping diagrams of target gene expression at the level of cell clusters were obtained by the FeaturePlot function of R language. The mol2 files of the active ingredients of PL were obtained using the PubChem database (https://pubchem.ncbi.nlm.nih.gov/). The PDB files of the molecular structures of the target proteins were obtained using the RCSB PDB database (https://www.rcsb.org/). PyMOL and AutoDock Tools software were used to dehydrate, hydrogenate, and delete the original ligand of the target protein, save it as a pdbqt file for the receptor, and save the mol2 file of PL active ingredient as a pdbqt file for the ligand. The center of the binding pocket is centered on the ligand, which contains the smallest possible area for the ligand to bind, and was obtained by analysis with AutoDock Tools software. Molecular docking was achieved by the AutoDock Vina software, using the Vina force field for optimisation and binding energy calculations. Docking parameters are as follows: exhaustiveness is 8, and num_modes is 9. Root mean squared deviation (RMSD) analysis was performed on the docking results, and interaction pattern analysis was performed using PyMOL.

### 2.6. Prognostic Analysis and Immunohistochemical Validation

**Figure 1:**
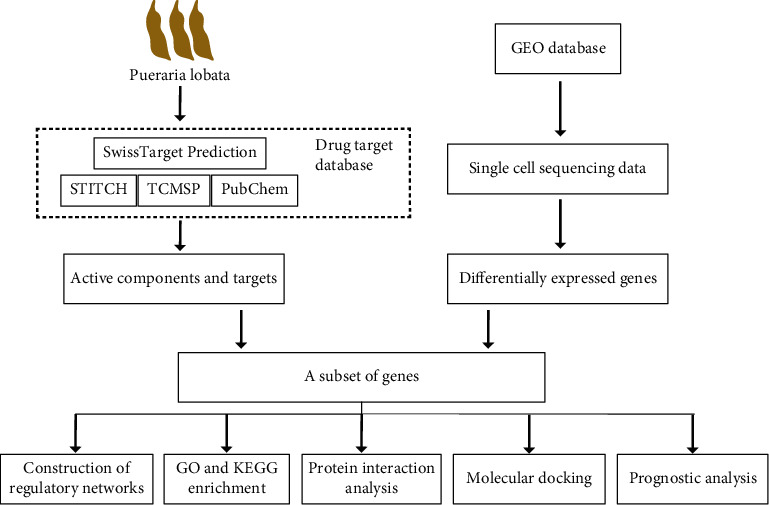
Flowchart.

R language was used to obtain the data of 494 patients with PCa with complete clinical and transcriptome data from the cBioPortal database (https://www.cbioportal.org/). Progression-free survival (PFS) was analyzed by the univariate Cox proportional risk analysis and Kaplan–Meier analysis. Gene expression was analyzed by Student's *t*-test. From the online database Protein Atlas (https://www.proteinatlas.org/), the immunohistochemical microscopic images of genes were obtained and exported for visualization using image processing software Adobe Illustrator. The specific flow is shown in Figure 1.

### 2.7. Statistical Analysis

All statistical analyses and graphical representations were calculated using the R software version 4.0.4 and corresponding packages. Student's *t*-test was used to evaluate whether a significant difference existed between the two groups. The correlation between gene expression levels and PFS in patients with PCa was analyzed by the univariate Cox proportional risk analysis and Kaplan–Meier curve analysis. *P* < 0.05 was considered statistically significant.

## 3. Results

### 3.1. Single-Cell Data Integration and Differential Gene Screening

**Figure 2:**
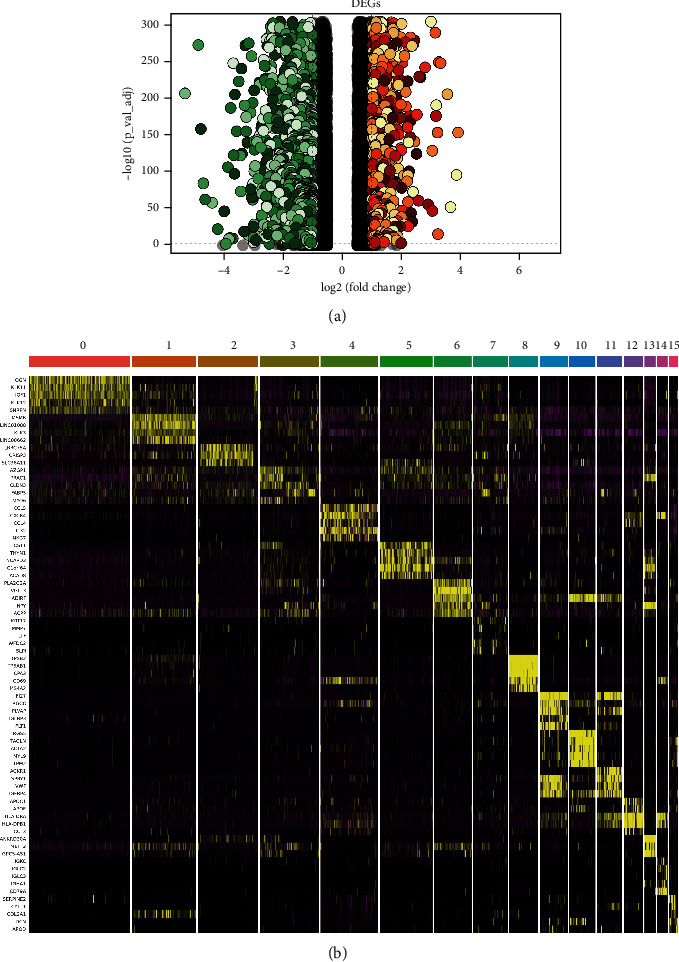
Differential genes. (a) Differential gene volcano plot, where red indicates upregulation and green indicates downregulation; the darker the color is, the greater the absolute value of log2FC. (b) Heat map of top five differential genes in 16 groups.

**Figure 3:**
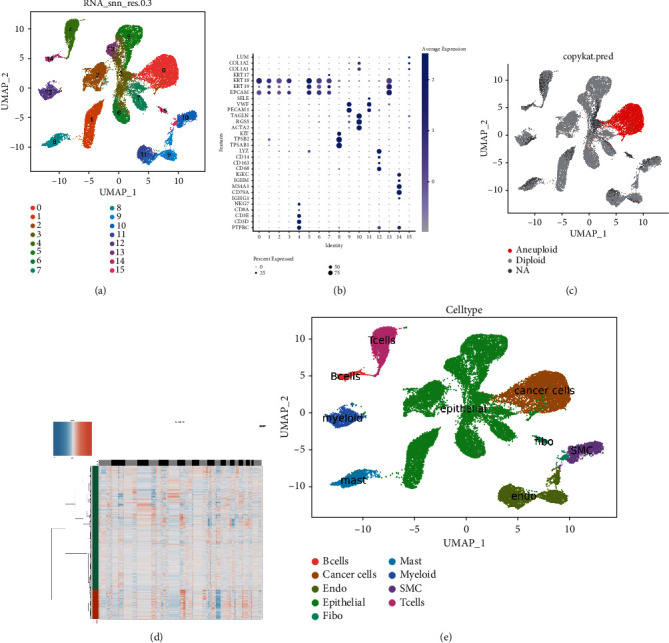
Definition of cell groupings. (a) Clustering map of single cell at 0.3 resolution. (b) Bubble map of marker genes in different clusters. (c) UMP map of CNV, where red dots indicate noninteger copies of cell genes. (d) CNV chromosome heat map. (e) UMP map defining cell populations.

After quality control, a single-cell matrix consisting of 33,602 cells and 23,698 genes was obtained and divided into 16 cell clusters (Figure. S1). DEGs were screened by FindAllMarkers package in R language. A total of 1,675 genes were obtained at the single-cell level (Figure 2). Sixteen cell clusters were defined according to marker genes (Table S1 and Figures 3(a) and 3(b)), and tumor cells were defined according to CNV results (Figures 3(c) and 3(d)). Finally, B cells, cancer cells, endothelial cells, epithelial cells, fibroblasts, mast cells, myeloid cells, smooth muscle cells, and T cells totaling nine cell types were obtained (Figure 3(e)).

### 3.2. Construction of Drug Component–Target Gene–Pathway Network Diagram

**Figure 4:**
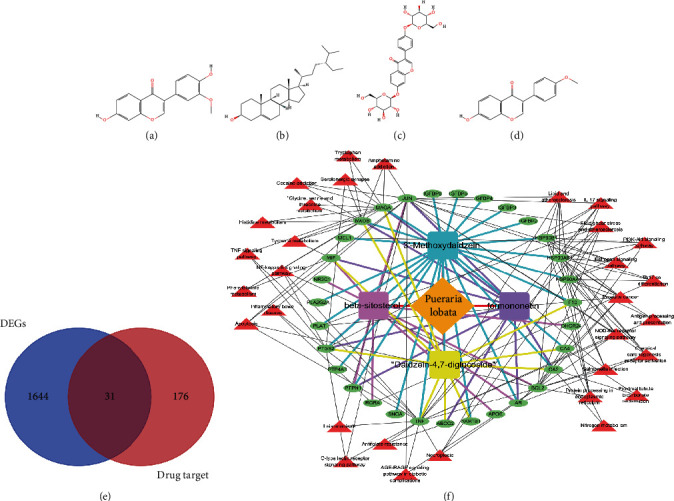
Drug–active monomer–target pathway network. (a–d) The four active components of PL were 3′-methoxydaidzein, beta-sitosterol, daidzein-4,7-diglucoside, and formononetin. (e) Venn diagram of differential genes and drug targets. (f) Drug–active monomer–target pathway network diagram. The yellow–brown prism is the PL, the rectangle represents the four active components, the green ellipse is the intersection target gene, and the red triangle is the KEGG pathway.

Four active components of PL were obtained by TCMSP: 3′-methoxydaidzein, beta-sitosterol, daidzein-4,7-diglucoside, and formononetin (Table 1 and Figures 4(a)–4(d)). Then, the active components' targets were obtained by using TCMSP, STITCH, SwissTargetPrediction, and PubChem databases. After the duplication was deleted, 207 PL targets were obtained. The intersection of drug target and DEGs resulted in 31 target genes (Figure 4(e)). According to the corresponding relationship of target genes and pathway, the drug component–target pathway network was constructed (Figure 4(f)). We analyzed the topological parameters of the network (Table S2). Then, we constructed the core target network of the network, in which 13 core target genes were obtained (Figure S2).

### 3.3. Functional Enrichment Analysis and Protein Interaction Analysis

**Figure 5:**
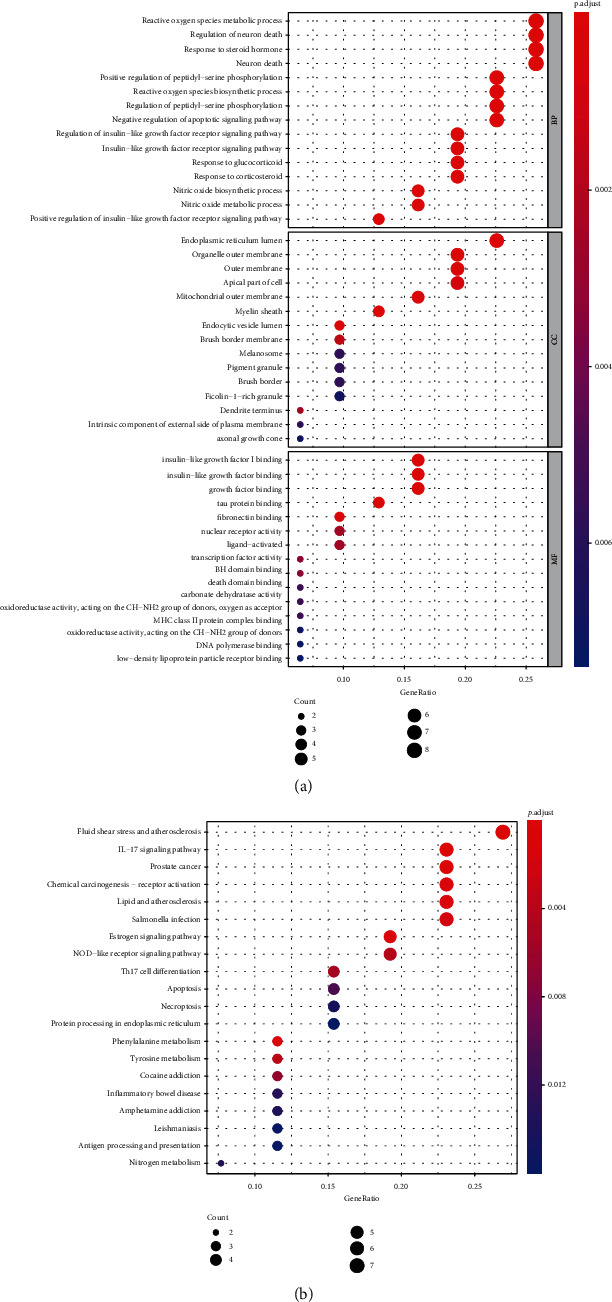
Functional and pathway enrichment analyses. (a) GO function enrichment bubble diagram, where BP represents the biological function, CC represents the cell component, and MF represents the molecular function. (b) KEGG pathway enrichment bubble diagram.

**Figure 6:**
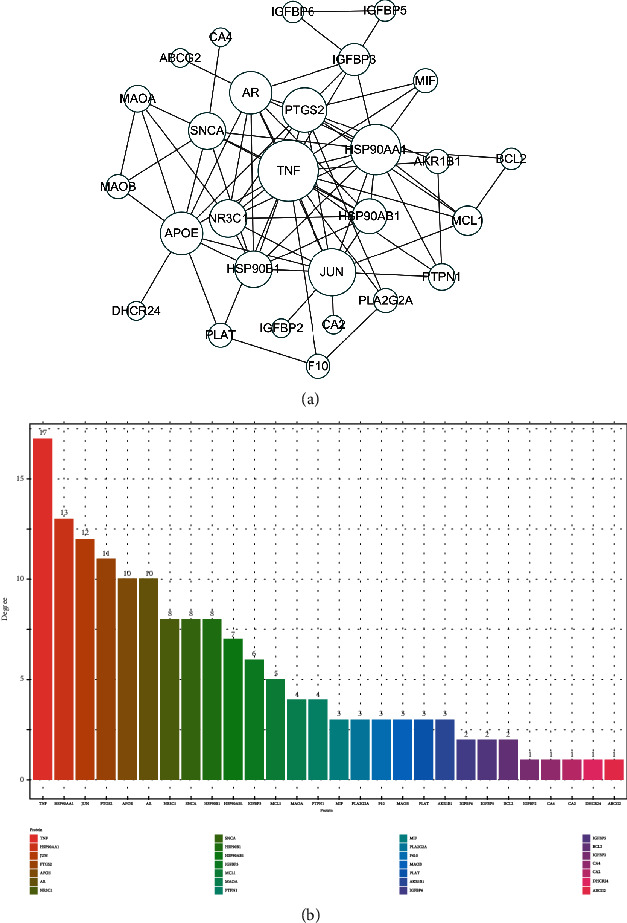
Protein interaction network. (a) Protein interaction loop diagram. (b) Histogram of protein interaction fraction.

The functional enrichment analysis of target genes and 362 bioinformatic expressions was obtained by GO enrichment analysis. The enriched expression of the top 15 comprehensive permutations was taken (Figure 5(a)), including metabolic processes of reactive oxygen species, response to steroid hormones' endoplasmic reticulum lumen, mitochondrial membrane, growth factor binding, tau protein binding, and fibronectin. A total of 31 signaling pathways were screened by KEGG enrichment analysis and binding. Top 10 enrichment analysis of KEGG was performed (Figure 5(b)), including fluid shear stress and atherosclerosis, IL-17 signaling pathway, PCa, chemoattractant-receptor activation, and estrogen signaling pathway. The top 10 proteins of the protein interaction network were *TNF*, *HSP90AA1*, *JUN*, *PTGS2*, *APOE*, *AR*, *NR3C1*, *SNCA*, *HSP90B1*, and *HSP90AB1* (Figure 6). Most of them were enriched in the above functions and pathways.

### 3.4. Single-Cell Data Target Gene Expression and Molecular Docking

**Figure 7:**
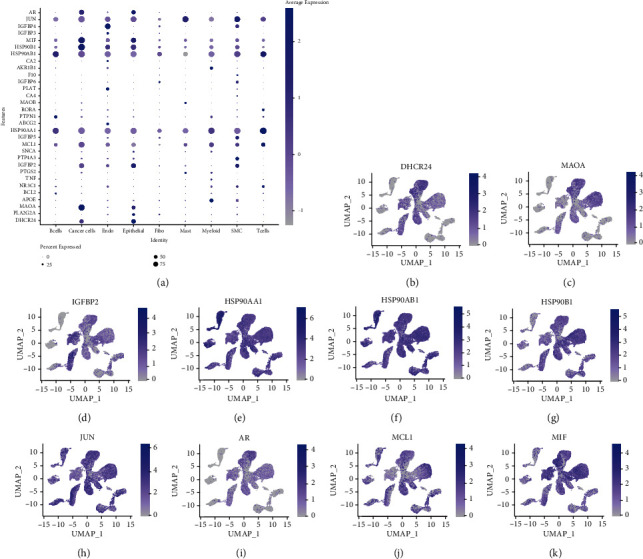
Expression of target genes in each cell population. (a) Bubble map of target gene expression. (b–k) Coordinate mapping of *DHCR24*, *MAOA*, *IGFBP2*, *HSP90AA1*, *HSP90AB1*, *HSP90B1*, *JUN*, *AR*, *MCL1*, and *MIF*.

**Figure 8:**
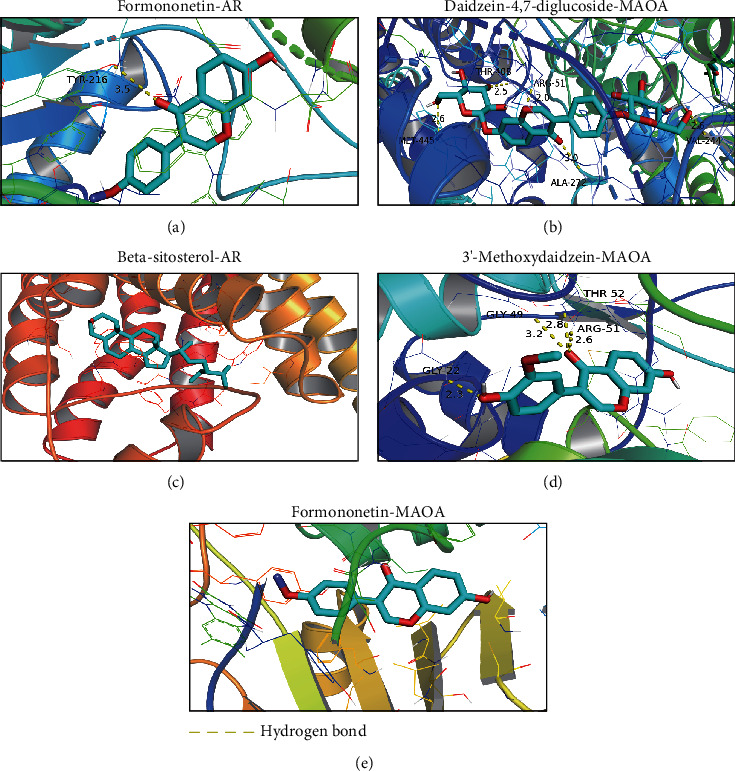
Molecular docking results.

The single-cell data were integrated with the target genes by the Seurat package in R language, and the expression levels of 31 target genes on nine cell populations were obtained (Figure 7(a)). The overall expression levels of *DHCR24*, *MAOA*, *IGFBP2*, *HSP90AA1*, *HSP90AB1*, *HSP90B1*, *JUN*, *AR*, *MCL1*, and *MIF* genes were relatively high, and the expression in each cell group was obtained by coordinate mapping (Figures 7(b)–7(k)). The coordinate maps of the remaining target genes were presented in Supplementary Figures (Figure. S3). In cancer cells, *AR*, *MIF*, *HSP90B1*, and *MAOA* were expressed in more than 50% of cells at a high level relative to other genes. Therefore, these four proteins were selected as the targets for molecular docking, and the results showed that the docking effect of the active monomer and the spatial conformation of the target protein was good (the top five docking scores/binding energies are shown in Figure 8, and the rest of the docking results were shown in the supplementary Figure. S4). Docking score/binding energy was good (Table 2). The main force is hydrogen bond; *π*-sigma and van der Waals forces are not found in the current study (Figure 8 and Figure. S4).

### 3.5. Prognostic Analysis Results and Immunohistochemical Validation

**Figure 9:**
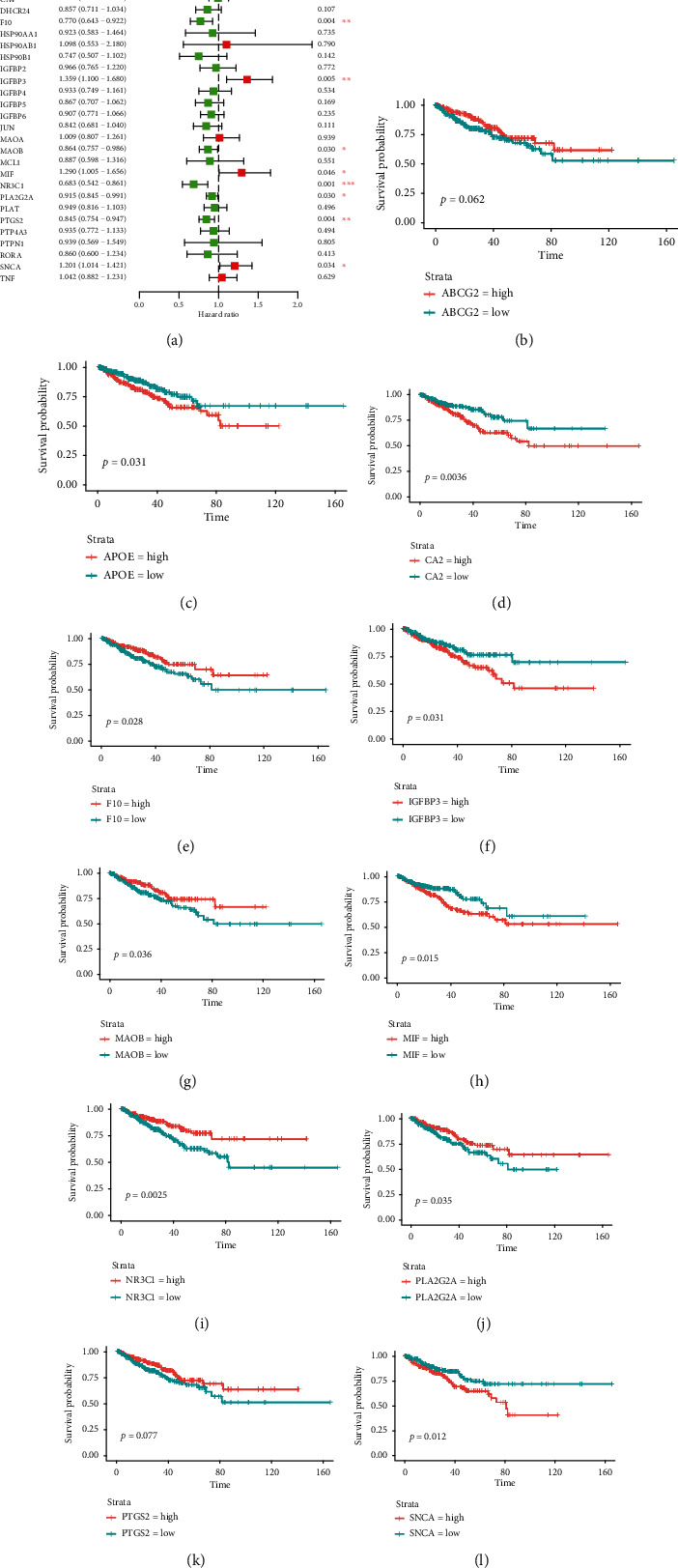
Survival analysis. (a) Forest map of the univariate Cox proportional risk analysis for 31 target genes. (b–l) Curves of 11 genes, *ABCG2*, *APOE*, *CA2*, *F10*, *IGFBP3*, *MAOB*, *MIF*, *NR3C1*, *PLA2G2A*, *PTGS2*, and *SNCA*.

**Figure 10:**
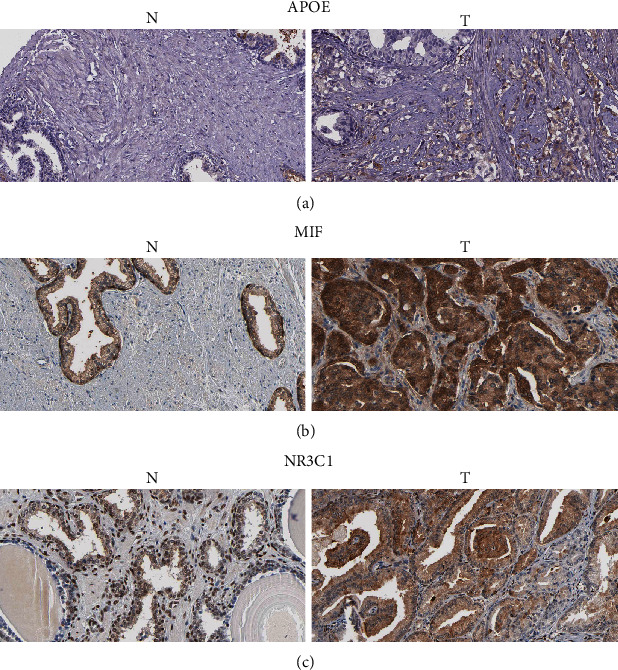
Immunohistochemistry. (a) Immunohistochemical plots of *APOE* in PCa and normal tissues. (b) Immunohistochemical plots of *MIF* in PCa and normal tissues. (c) Immunohistochemical plots of *NR3C1* in PCa and normal tissues.

The univariate Cox proportional risk analysis of 31 target genes identified 11 genes, *ABCG2*, *APOE*, *CA2*, *F10*, *IGFBP3*, *MAOB*, *MIF*, *NR3C1*, *PLA2G2A*, *PTGS2*, and *SNCA*, were associated with PFS (*P* < 0.05). The Kaplan–Meier survival analysis obtained *APOE*, *CA2*, *F10*, IGFBP3, *MAOB*, *MIF*, *NR3C1*, *PLA2G2A*, and *SNCA* differed in PFS (*P* < 0.05), but *ABCG2* and *PTGS2* showed no statistically significant difference in PFS (*P* > 0.05) (Figures 9(b)–9(l)). The grouping expression results showed that *APOE*, *CA2*, *IGFBP3*, *MIF*, *F10*, *NR3C1*, and *PTGS2* were different between the groups with and without disease progression (*P* < 0.05) (Figure. S5). Together with the univariate Cox proportional risk analysis, Kaplan–Meier survival analysis, and subgroup expression, six genes, *APOE*, *CA2*, *IGFBP3*, *MIF*, *F10*, and *NR3C1*, were obtained to predict PFS. *APOE*, *MIF*, and *NR3C1* were found differentially expressed in PCa by Protein Atlas and with elevated expression compared with normal tissues (Figures 10(a)–10(c)).

## 4. Discussion

Currently, the treatment of PCa mainly consists of various physical and chemical methods. Minimally invasive ablation, radiotherapy, or radical PCa resection can be used to treat PCa in the early, middle, or localized stages [12–14]. Chemotherapy is always the final option as the disease progresses. However, resistance is the cause of chemotherapy failure in 90% of patients with cancer [15]. Among the six drugs approved by the FDA for the treatment of metastatic drug-resistant PCa, the average improvement in overall survival is only 4.8 months, and drug resistance likely is the main cause [16]. In addition, chemotherapy often induces various serious side effects. Therefore, new treatments that can improve efficacy and reduce side effects need to be sought. PL is one of the TCMs, mainly composed of isoflavones, flavonoids, flavonols, fragrant plum alcohols, and other compounds, among which the main biologically active components are isoflavones, also known as phytoestrogens [17, 18]. Previous studies demonstrated that isoflavones have a significant role in the treatment of hormone-dependent tumors [19–21]. PCa is one of hormone-dependent tumors, and the therapeutic effect of PL in PCa remains unclear. Therefore, this paper explored the potential role of PL in PCa from the perspective of single-cell network pharmacology.

In this paper, four active compounds of PL were screened: 3′-methoxychasteel, *β*-sitosterol, chasteberry-4,7-diglucoside, and formonetin. The beta-sitosterol inhibited the growth of PC cell lines in several ways, including inhibition of proliferation, apoptosis, and suppression of NF-*κ*B activity. Beta-sitosterol also inhibited migration and invasion and downregulated markers of the epithelial-mesenchymal transition [22]. Formonetin inhibits cell proliferation, tube formation, and cell migration and interferes with MYC and STAT3 proteins via the RAS/ERK and JAK1/STAT3 pathways to suppress PD-L1 protein expression thereby promoting tumor cell apoptosis [23]. Evidence suggests that the active constituents of Pueraria lobata have multipathway, multitargeted antitumor effects, which provides clues for us to develop target exploration in prostate cancer. The results showed that PL exerted potential interventions on PCa through 31 target genes and 31 signaling pathways corresponding to the above compounds. The results of functional enrichment analysis showed that PL played a pharmacological role in the treatment of PCa by regulating the metabolic processes of reactive oxygen species, response to steroid hormones, and oxidative stress as well as IL-17 signaling pathway, PCa, and estrogen signaling pathway, which involved biological processes such as sensitivity to steroid hormones, cellular metabolism, cytokines, and gene transcription. The results suggested that PL can intervene in PCa by participating in various biological processes and signaling pathways. IL-17 can promote epithelial mesenchymal transition and tumor cell invasion by inducing the expression of *MMP7* in PCa cells, disrupting the E-calmodulin/*β*-linked protein complex, and releasing *β*-linked protein [24]. Estrogen can activate the SRC and PI3K/AKT pathways through binding receptors and promote the expression of nonphosphorylated *β*-linked proteins, thereby enhancing PC-3 cell proliferation, migration, invasion, and tumor formation [25]. The findings suggested that PL may intervene in PCa by regulating signaling pathways and biological processes such as interleukin-17 and estrogen.

Based on marker genes, nine cell populations were obtained, including cancer cells, to explore the potential role of PL in the treatment of PCa at the single-cell level. *AR*, *MIF*, *HSP90B1*, and *MAOA* were expressed in more than 50% of cancer cells with high expression levels and may have potential therapeutic targets. In almost all PCa patients, the androgen receptor (AR) was the main driver of tumor cell genesis and development. It was activated and translocated to the nucleus to bind to androgen response elements in DNA and recruit regulatory factors or transcription factors that mediated target gene transcription to regulate biological processes such as cell proliferation, apoptosis, migration, invasion, and differentiation [26–28]. Our paper found that 3′-methoxydaidzein, beta-sitosterol, and formononetin in PL could act as AR in PCa possibly with potential antitumor effects. Previous studies found that *MIF* can promote PCa growth and metastasis by upregulating *MAPK* and *CXCR7* expressions, thus activating the PI3K-AKT signal transduction pathway [29]. HSP90B1 downregulation mediated the PI3K/AKT/mTOR pathway to inhibit tumor growth in vitro and in vivo [30]. In addition, HSP90B1 overexpression promoted the proliferation, migration, and invasion of bladder and breast cancer cells in vitro [31, 32]. MAOA promoted PCa metastasis by regulating downstream ROS and Twist1 pathways that mediated Shh/Gli signaling to activate YAP1 transcription in concert with AR to induce epithelial–mesenchymal transition and tumor–stromal cell interactions [33–35]. *AR*, *MIF*, *HSP90B1*, and *MAOA*, as the targets of PL, exerted antitumor effects by regulating related pathways, suggesting that PL can interfere with tumorigenesis and progression through multicomponent, multitarget, and multipathway mechanisms. In addition, molecular docking experiments showed that the active monomers of PL had a spatial conformational docking effect with the proteins of the four corresponding target genes with good docking scores/binding energy, suggesting that PL may affect the protein activity and the regulation of its downstream pathways by directly binding to the active centers of the proteins. *APOE*, *CA2*, *IGFBP3*, *MIF*, *F10*, and *NR3C1* were obtained for the prediction of PFS in PCa by the univariate Cox proportional risk analysis and Kaplan–Meier survival analysis of target genes. Protein Atlas verified that *APOE*, *MIF*, and *NR3C1* were expressed in tumor tissues at different levels. The findings suggested that PL may intervene in the clinical prognosis of PCa by acting on the corresponding targets.

This study may provide clues to the search for potential therapeutic targets and drugs for prostate cancer and refine our understanding of the efficacy of Pueraria lobata and its active ingredients. The limitation of this study is that the components of herbal medicine are complex and diverse in their therapeutic effects, and it is uncertain which combinations are at play, and more evidence is needed.

## 5. Conclusion

In this paper, the drug–active ingredient–target pathway network of Chinese herbal medicine PL was constructed at the single-cell level of PCa by network pharmacology combined with single-cell sequencing technology. The findings revealed that its action on *AR*, *MIF*, *HSP90B1*, and *MAOA* genes regulated several biological processes and related signaling pathways to interfere with the occurrence and development of PCa while its action on *APOE*, *CA2*, *IGFBP3*, *MIF*, *F10*, and *NR3C1* genes was also important in interfering with clinical prognostic regression. The multiple drug–active component–target pathway axis constructed in this paper provided a new direction for exploring the therapeutic and prognostic targets of PCa. The above results were initially validated in molecular docking experiments and Protein Atlas, and the specific related molecular mechanisms need to be investigated further through experiments.

## Figures and Tables

**Table 1:** Active components of PL.

MOL_ID	Molecule_name	ob	dl
MOL003629	Daidzein-4,7-diglucoside	47.275	0.674
MOL000358	Beta-sitosterol	36.914	0.751
MOL002959	3′-Methoxydaidzein	48.569	0.242
MOL000392	Formononetin	69.674	0.212

**Table 2:** Molecular docking results.

Active ingredients	Target protein	Docking score/binding energy	RMSD
3′-Methoxydaidzein	MAOA	-8.3	0.888
3′-Methoxydaidzein	MIF	-6.3	0.432
3′-Methoxydaidzein	AR	-5.4	0.371
3′-Methoxydaidzein	HSB90B1	-7.2	0.001
Formononetin	MAOA	-8.1	<0.001
Formononetin	MIF	-5.8	<0.001
Formononetin	AR	-10.8	<0.001
Daidzein-4,7-diglucoside	MAOA	-10.5	3.131
Daidzein-4,7-diglucoside	MIF	-5.8	1.273
Beta-sitosterol	AR	-9.2	0.222

Note: docking score/binding energy > −4 kcal/mol means weak binding capacity, −7 < docking score/binding energy < −4 means moderate binding capacity, and docking score/binding energy < −7 means strong binding ability. A smaller value of RMSD indicates a higher spatial overlap between the active monomer and the best bound conformation; that is, a smaller value means a higher chance of successful docking.

## Data Availability

Data is openly available in a public repository.

## Supplementary Materials

Supplementary Materials Supplementary forms and pictures are in supplementary files.
