# Supplementary material for: Pueraria lobata Potentially Treating Prostate Cancer on Single-Cell Level by Network Pharmacology and AutoDock: Clinical Findings and Drug Targets
Source: Comput Math Methods Med. 2022 Nov 21;2022:3758219. doi: 10.1155/2022/3758219 (PMC9705089; doi:10.1155/2022/3758219)
Supplement: Supplementary Materials — Supplementary forms and pictures are in supplementary files. [file 3758219.f1.zip › Figure.S4.pdf]

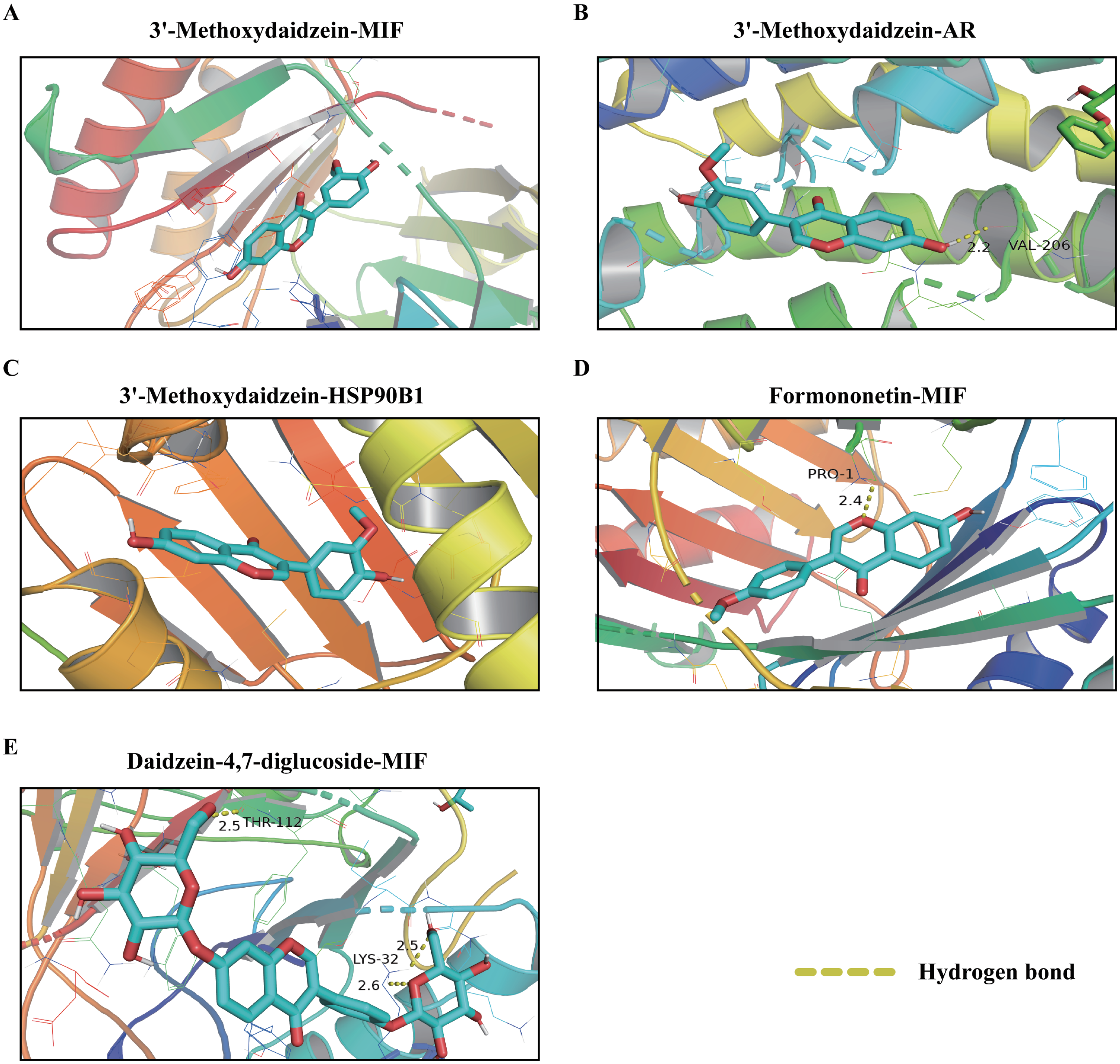
A
3'-Methoxydaidzein-MIF
B
3'-Methoxydaidzein-AR
2.2
VAL-206
C
3'-Methoxydaidzein-HSP90B1
D
Formononetin-MIF
PRO-1
2.4
E
Daidzein-4,7-diglucoside-MIF
2.5
THR-112
2.5
LYS-32
2.6
Hydrogen bond
