# Supplementary material for: Pueraria lobata Potentially Treating Prostate Cancer on Single-Cell Level by Network Pharmacology and AutoDock: Clinical Findings and Drug Targets
Source: Comput Math Methods Med. 2022 Nov 21;2022:3758219. doi: 10.1155/2022/3758219 (PMC9705089; doi:10.1155/2022/3758219)
Supplement: Supplementary Materials — Supplementary forms and pictures are in supplementary files. [file 3758219.f1.zip › Supplementary files.docx]

**Support data**

**Table S1 Marker genes**

| marker gene | cell type | References (PMID) |
| --- | --- | --- |
| PTPRC | T cell | 32938973 |
| CD3D | T cell | 33962884 |
| CD3E | T cell | 33962884 |
| CD8A | T cell | 32061950 |
| NKG7 | T cell | 34911739 |
| IGHG1 | B cell | 28474673 |
| CD79A | B cell | 33962884 |
| MS4A1 | B cell | 32061950 |
| IGHM | B cell | 28474673 |
| IGKC | B cell | 34185421 |
| CD68 | Macrophage | 28474673 |
| CD163 | Macrophage | 33962884 |
| CD14 | Macrophage | 28428369 |
| LYZ | Macrophage | 33962884 |
| TPSAB1 | Mast cell | 34185421 |
| TPSB2 | Mast cell | 34185421 |
| KIT | Mast cell | 28474673 |
| ACTA2 | smooth muscle cell | 33962884 |
| RGS5 | Smooth muscle cell | 33710643 |
| TAGLN | Smooth muscle cell | 33962884 |
| PECAM1 | endothelial cell | 33962884 |
| VWF | endothelial cell | 33962884 |
| SELE | endothelial cell | 35388143 |
| EPCAM | epithelial cell | 34185421 |
| KRT19 | epithelial cell | 34185421 |
| KRT18 | epithelial cell | 33032611 |
| KRT17 | epithelial cell | 34185421 |
| COL1A1 | fibroblast | 33962884 |
| COL1A2 | fibroblast | 33962884 |
| LUM | fibroblast | 33962884 |

**Table S2 Topological parameters of the drug component-target gene pathway network**

| Gene name | Closeness | Degree | Betweenness |
| --- | --- | --- | --- |
| JUN | 0.437086 | 18 | 562.1979 |
| TNF | 0.47482 | 17 | 603.3433 |
| HSP90AA1 | 0.44898 | 14 | 270.151 |
| HSP90AB1 | 0.44898 | 14 | 270.151 |
| BCL2 | 0.368715 | 14 | 226.0555 |
| MAOA | 0.425806 | 11 | 367.6731 |
| MAOB | 0.425806 | 11 | 367.6731 |
| PTGS2 | 0.44898 | 10 | 319.0033 |
| HSP90B1 | 0.420382 | 10 | 144.007 |
| AR | 0.420382 | 5 | 102.6115 |
| CA4 | 0.39521 | 5 | 136.6451 |
| CA2 | 0.39521 | 5 | 136.6451 |
| MIF | 0.360656 | 5 | 87.40261 |
| ABCG2 | 0.390533 | 4 | 50.93864 |
| PTPN1 | 0.409938 | 3 | 48.54309 |
| MCL1 | 0.385965 | 3 | 34.46205 |
| PLAT | 0.385965 | 3 | 20.96041 |
| RORA | 0.318841 | 3 | 16.0001 |
| F10 | 0.377143 | 2 | 4.091408 |
| AKR1B1 | 0.377143 | 2 | 4.091408 |
| PLA2G2A | 0.368715 | 1 | 0 |
| IGFBP2 | 0.368715 | 1 | 0 |
| PTP4A3 | 0.368715 | 1 | 0 |
| SNCA | 0.368715 | 1 | 0 |
| IGFBP5 | 0.368715 | 1 | 0 |
| IGFBP6 | 0.368715 | 1 | 0 |
| IGFBP3 | 0.368715 | 1 | 0 |
| IGFBP4 | 0.368715 | 1 | 0 |
| DHCR24 | 0.298643 | 1 | 0 |
| APOE | 0.298643 | 1 | 0 |
| NR3C1 | 0.298643 | 1 | 0 |

**
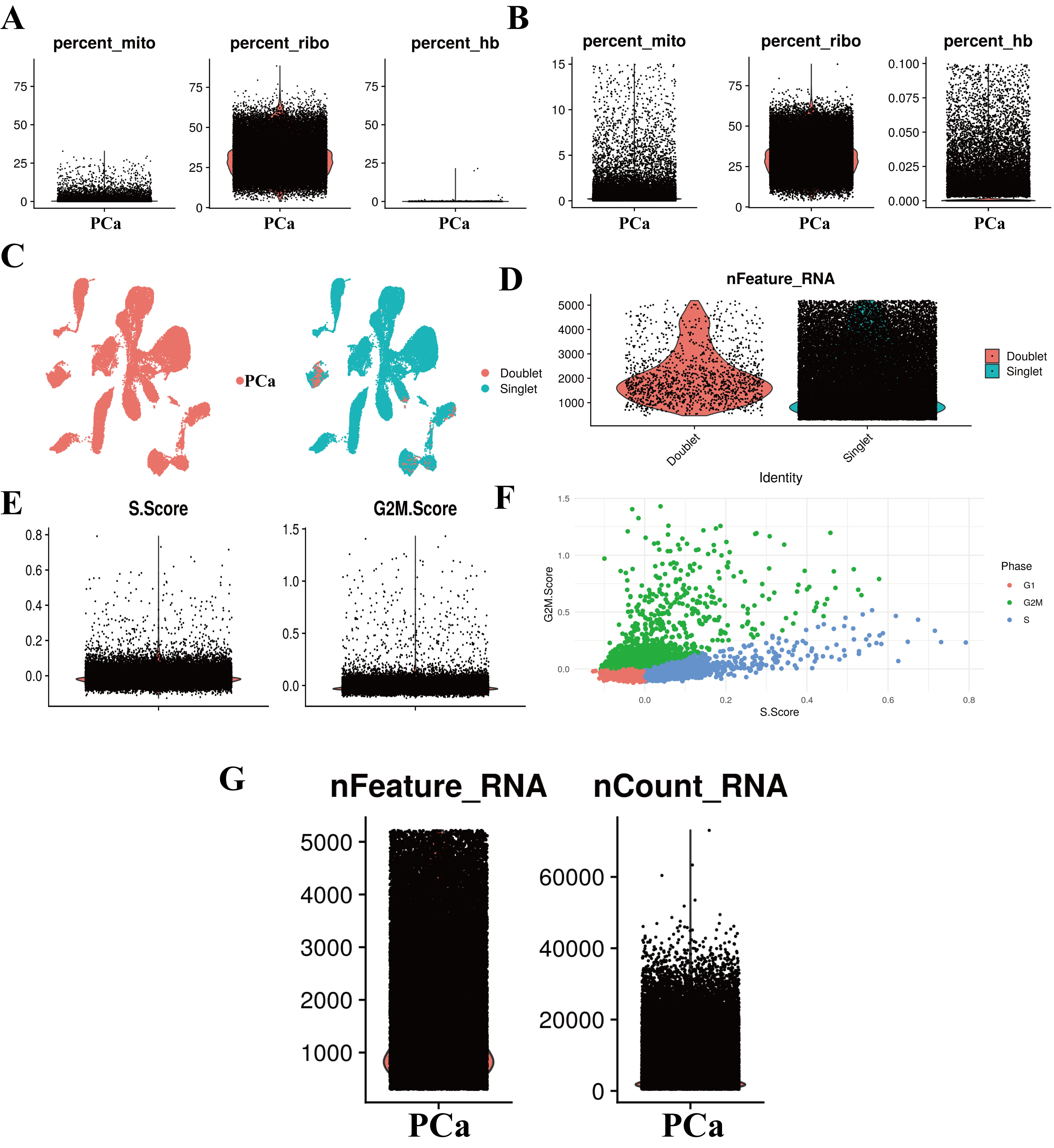
**

**Figure.S1 QC results of single cell data**

(A) Mitochondrial, ribosomal, and hemoglobin-related gene point plots before QC; (B) Mitochondrial, ribosomal, and hemoglobin-related gene point plots after QC; (C) UMP plots of duplicate sequenced cells, where the red dots on the right panel indicate duplicate sequenced cells; (D) Feature count point plots of sequenced cells; (E) Cell cycle score point plots; (F) Cell cycle distribution point plots; (G) Feature and count point plots.


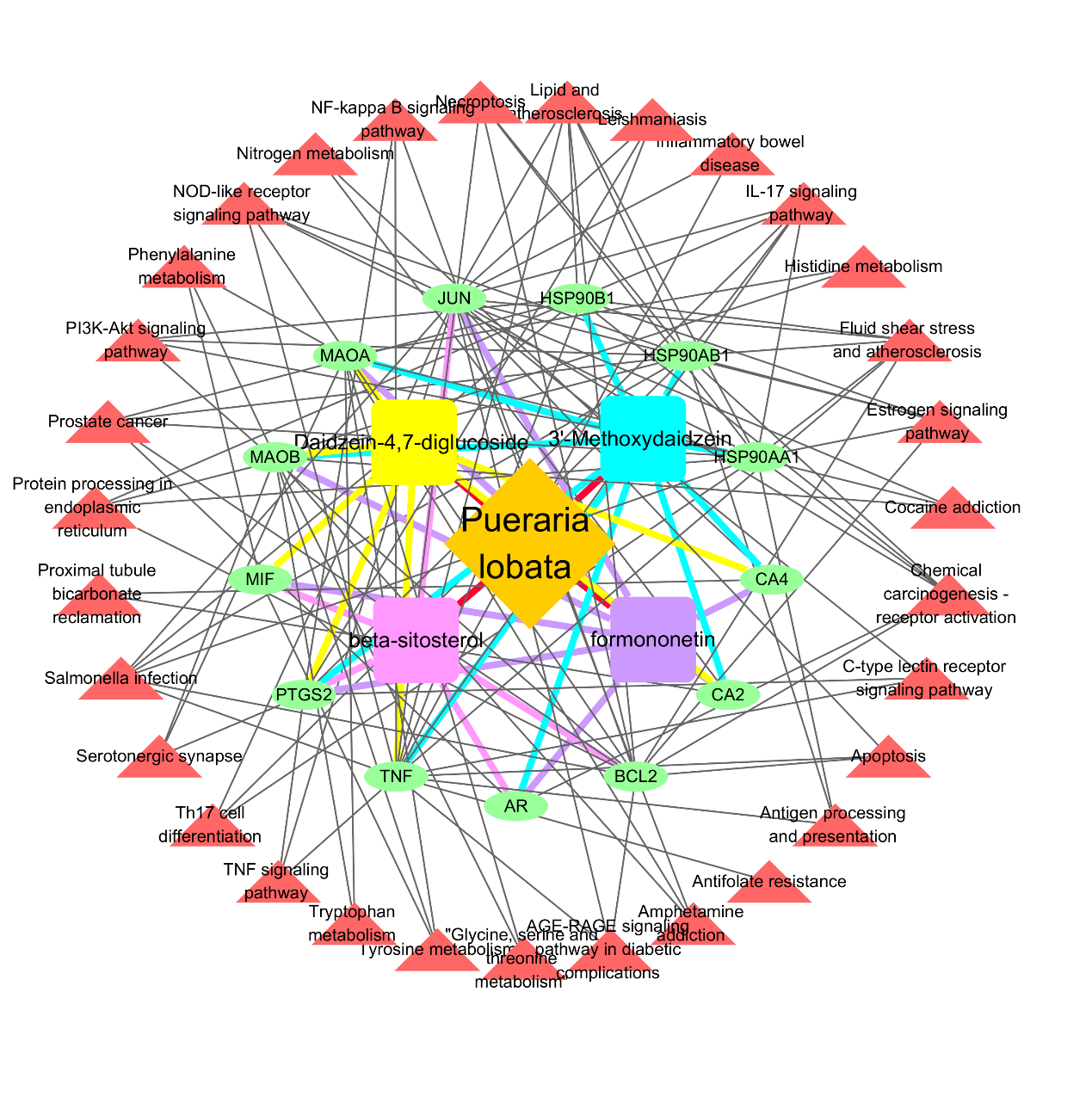


**Figure.S2 core targets networks**

Note: The yellow–brown prism is the PL, the rectangle represents the four active components, the green ellipse is the intersection target gene, and the red triangle is the KEGG pathway.





**Figure.S3 Expression of target genes in each cell population**

**
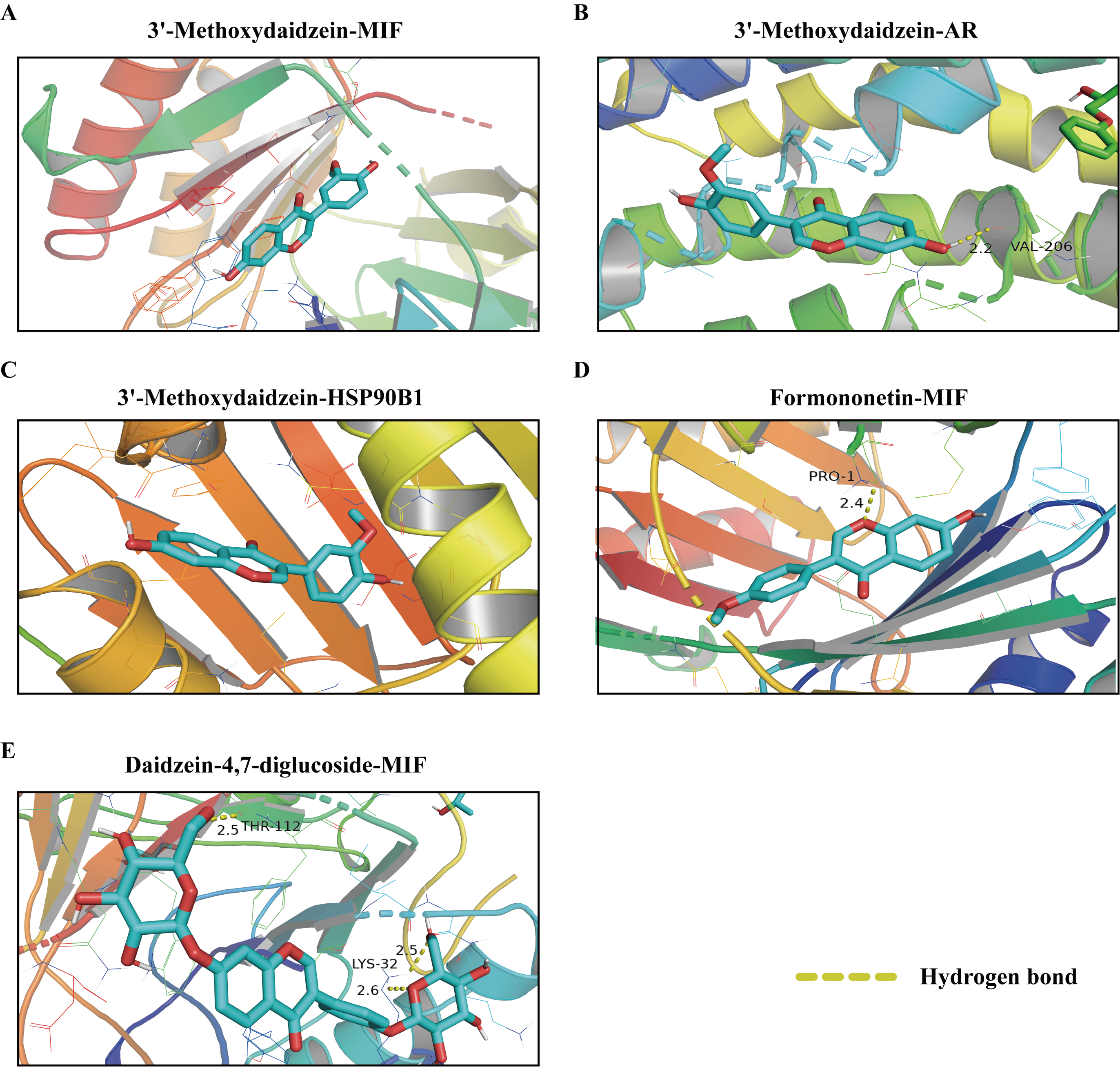
**

**Figure.S4 Molecular docking results**


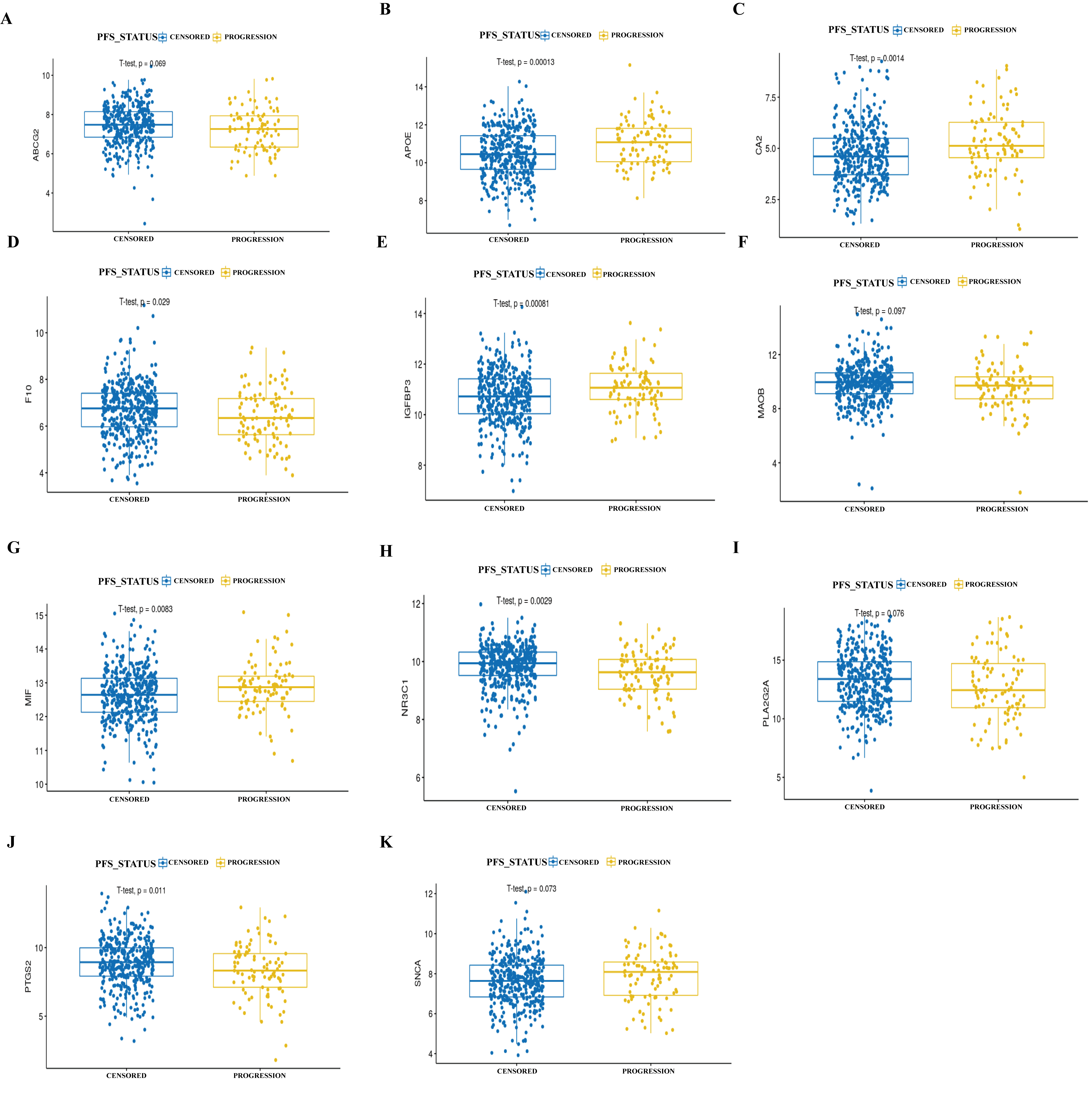


**Figure.S5 Gene grouping expression**
