# Supplementary figures and images for: Pueraria lobata Potentially Treating Prostate Cancer on Single-Cell Level by Network Pharmacology and AutoDock: Clinical Findings and Drug Targets
Source: Comput Math Methods Med. 2022 Nov 21;2022:3758219. doi: 10.1155/2022/3758219 (PMC9705089; doi:10.1155/2022/3758219)

**A**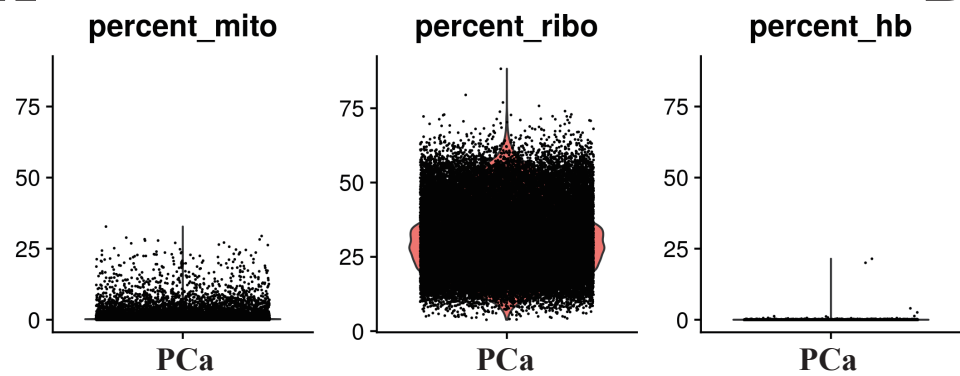**B**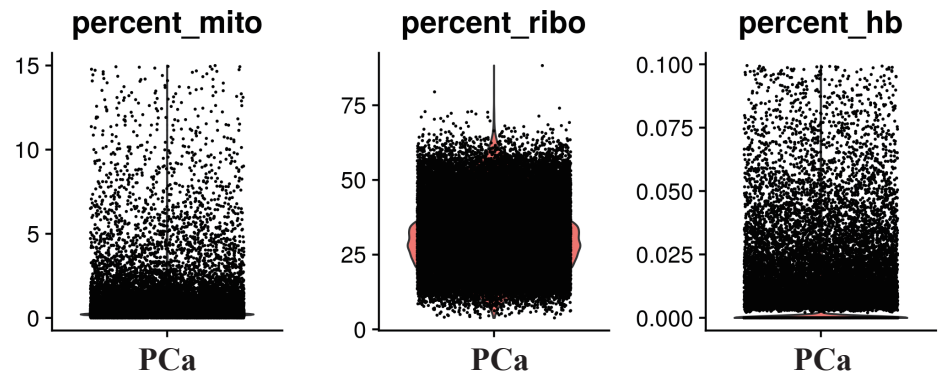**C**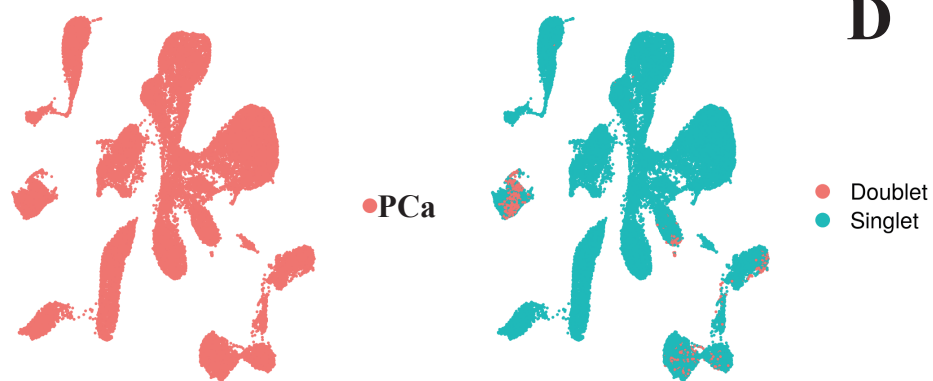**D**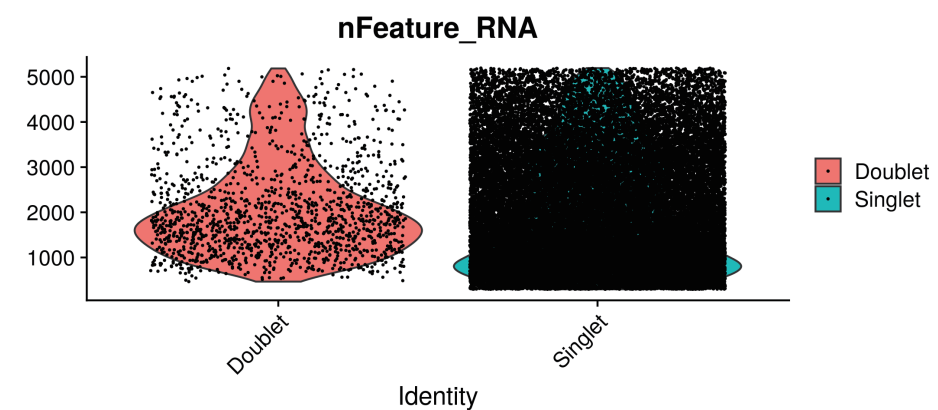**E**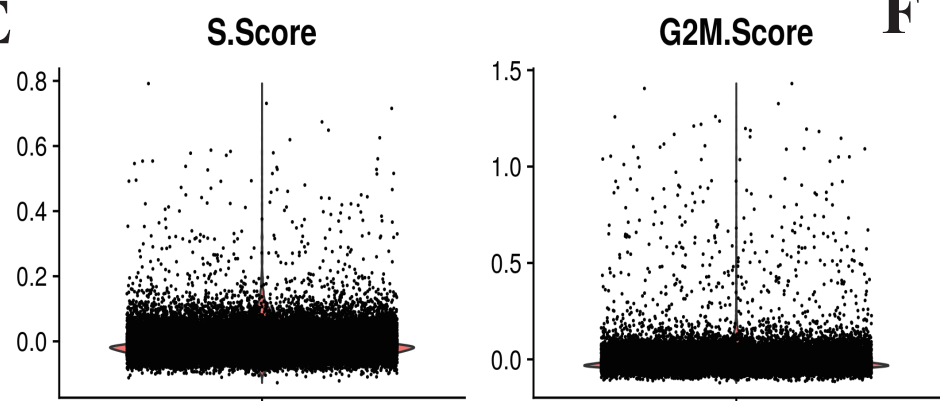**F**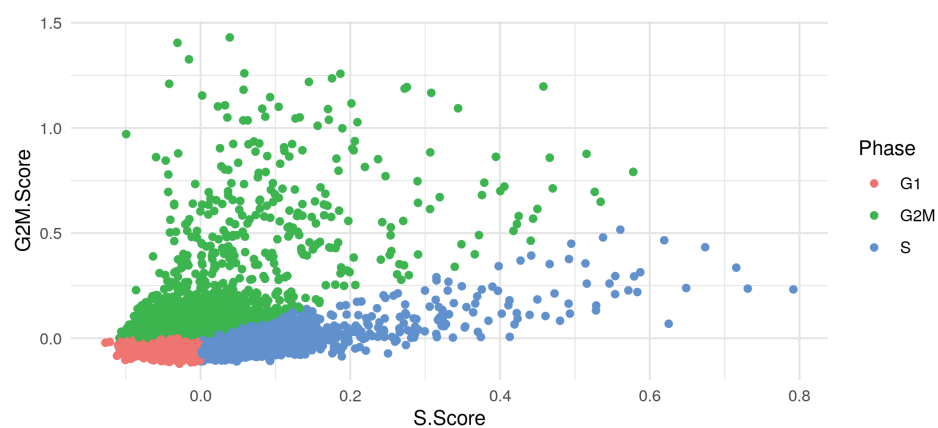**G**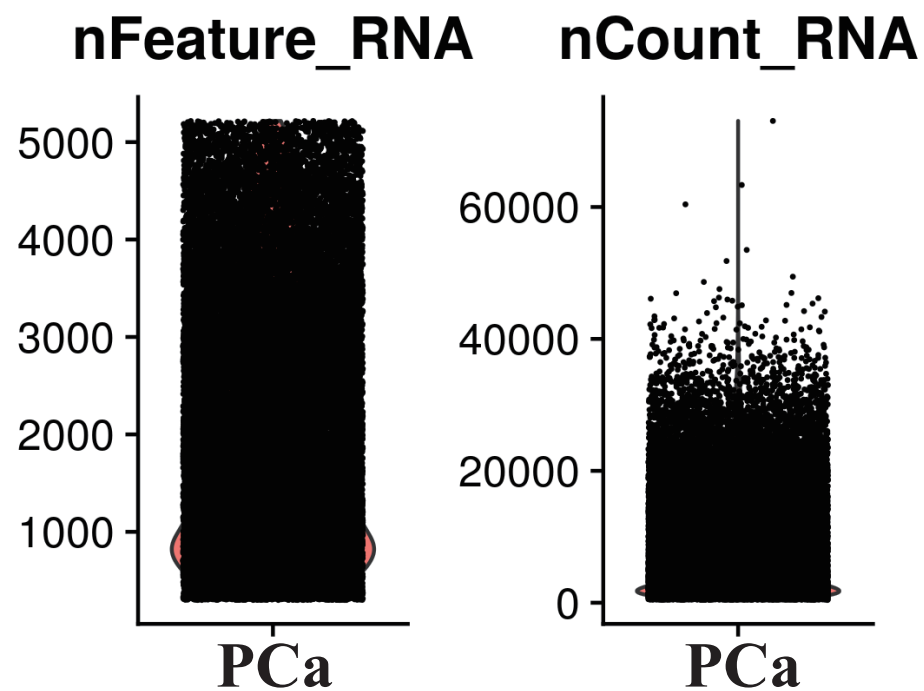

Supplement: Supplementary Materials — Supplementary forms and pictures are in supplementary files. [file 3758219.f1.zip › Figure.S1.pdf]

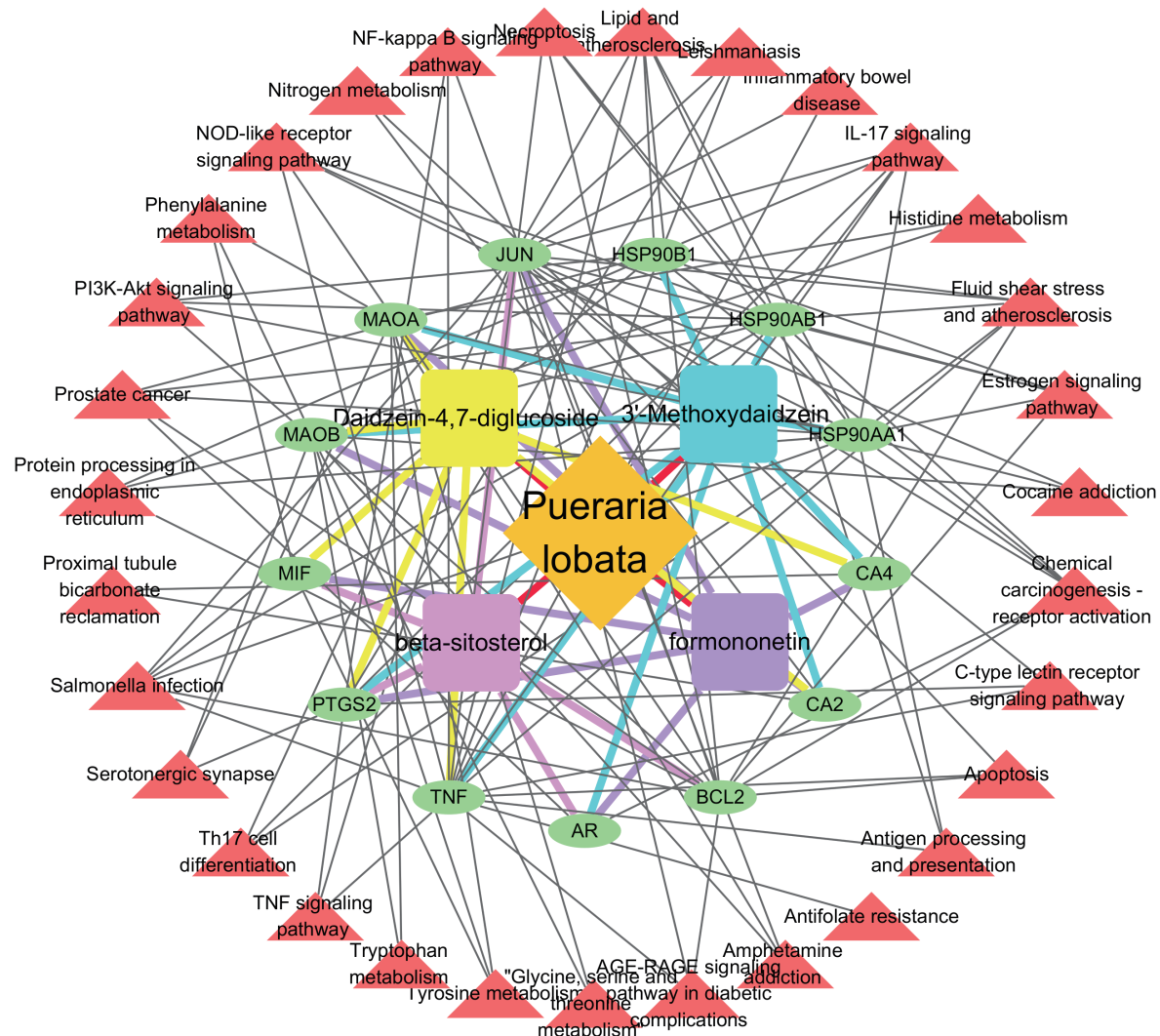

Supplement: Supplementary Materials — Supplementary forms and pictures are in supplementary files. [file 3758219.f1.zip › Figure.S2.pdf]

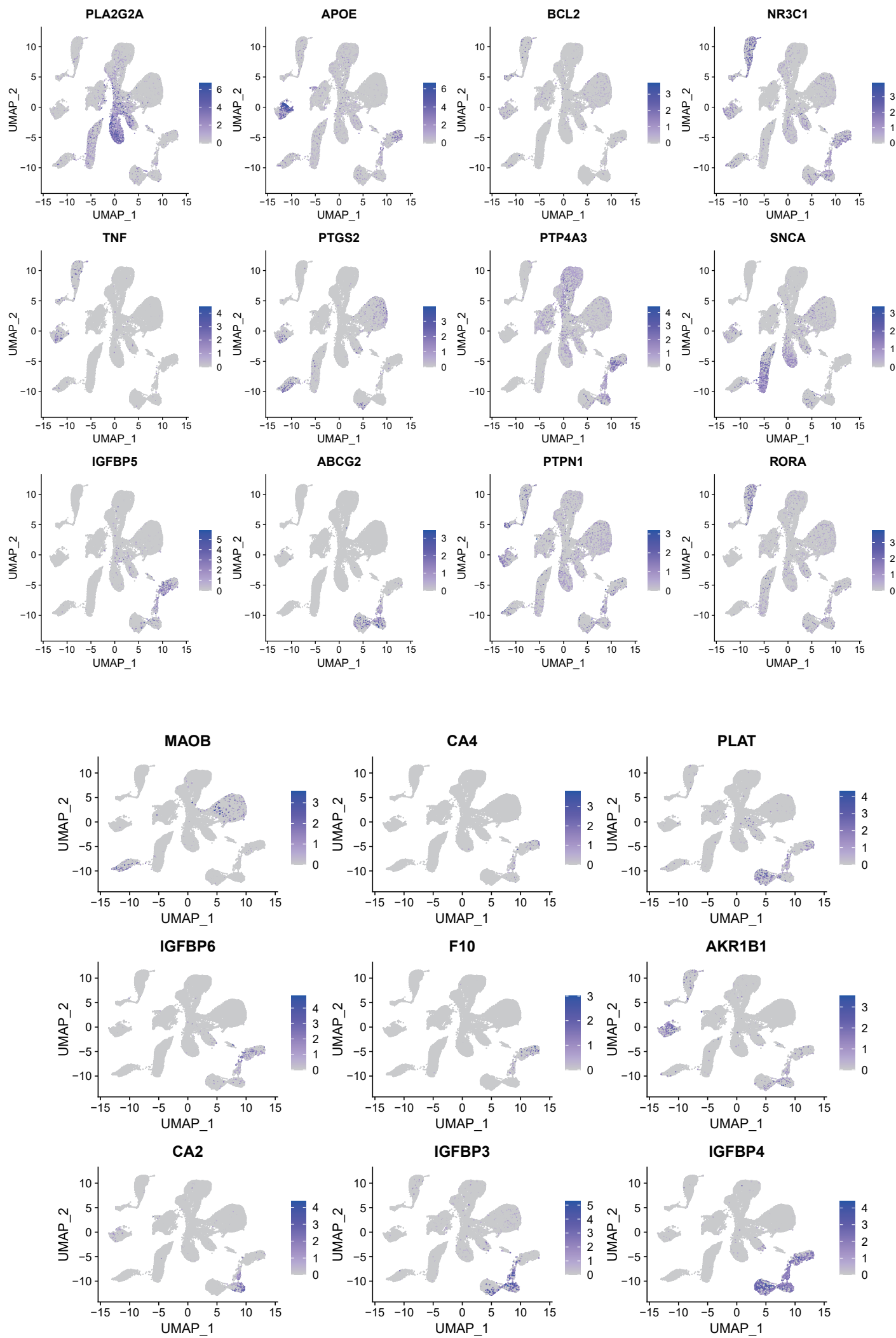

Supplement: Supplementary Materials — Supplementary forms and pictures are in supplementary files. [file 3758219.f1.zip › Figure.S3.pdf]

A

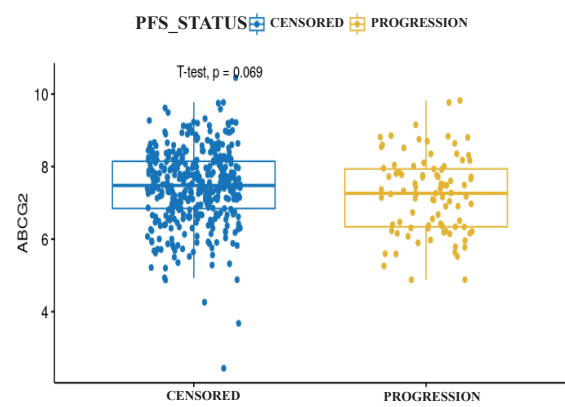

B

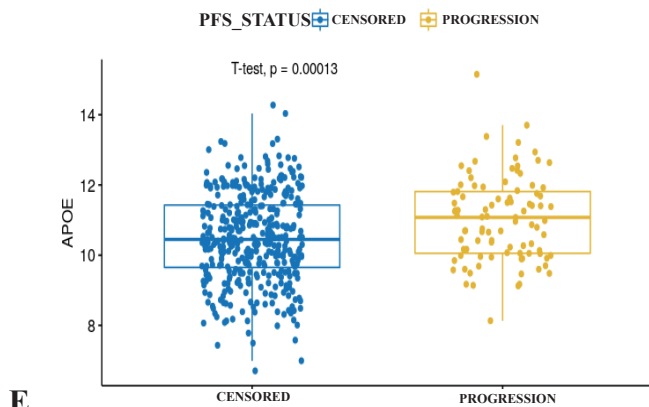

C

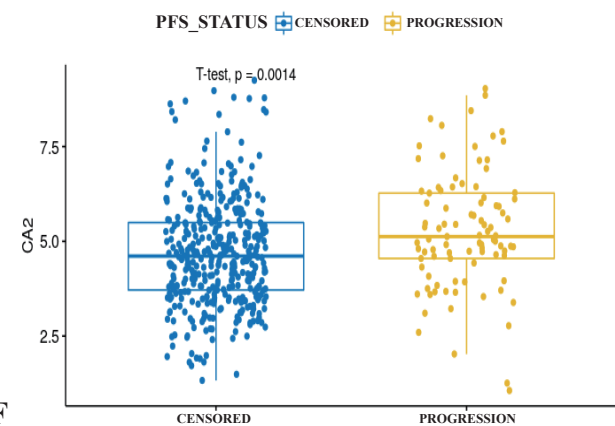

D

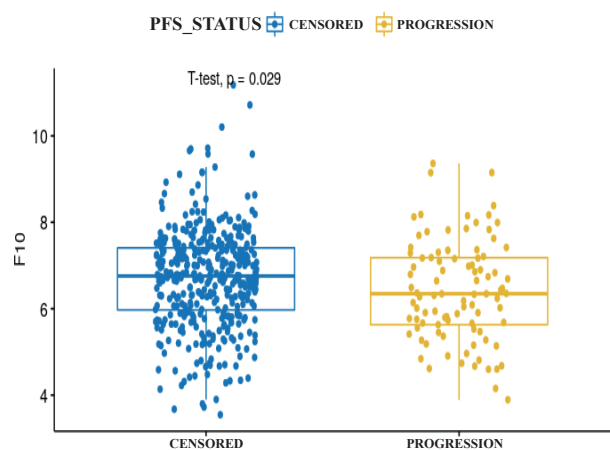

E

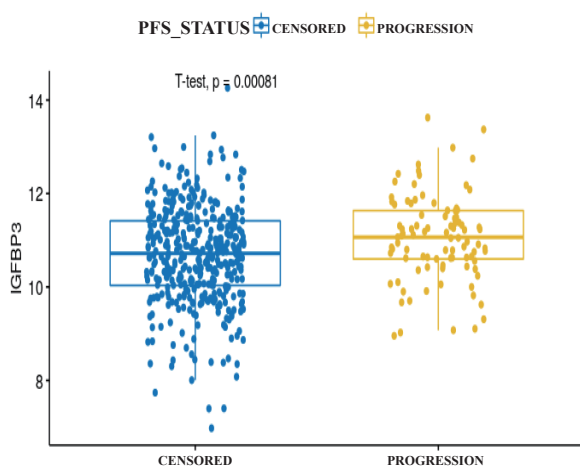

F

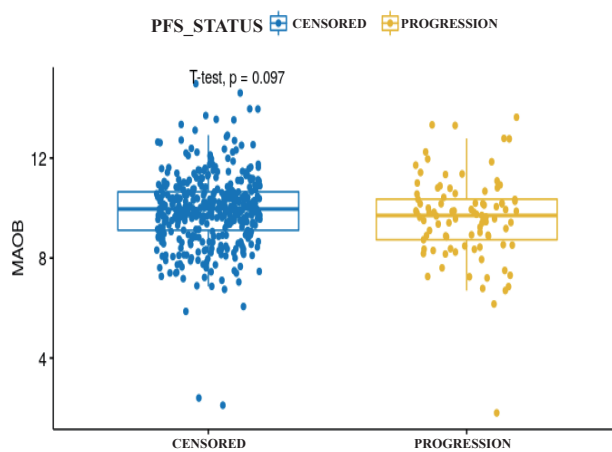

G

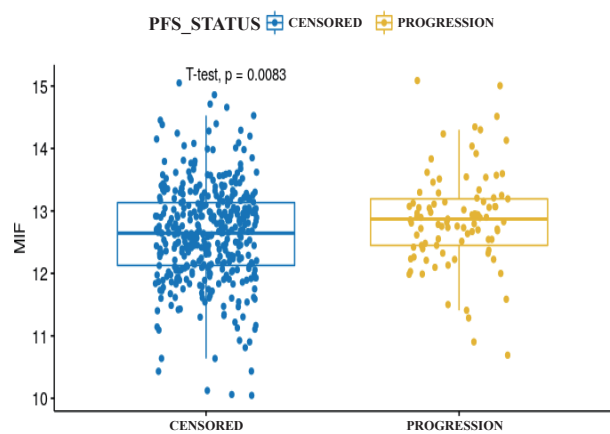

H

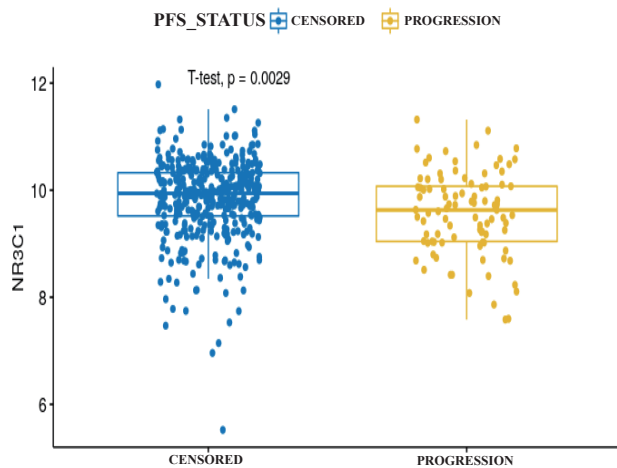

I

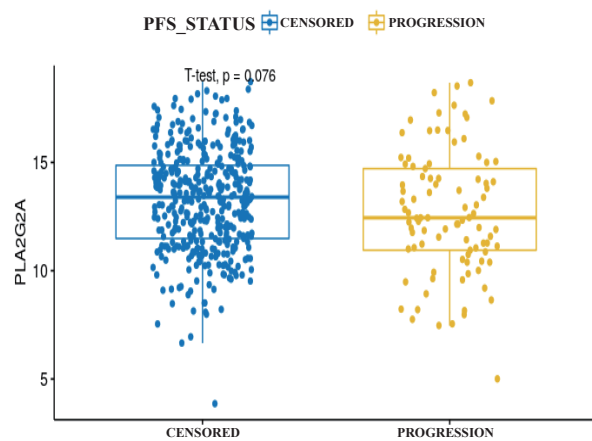

J

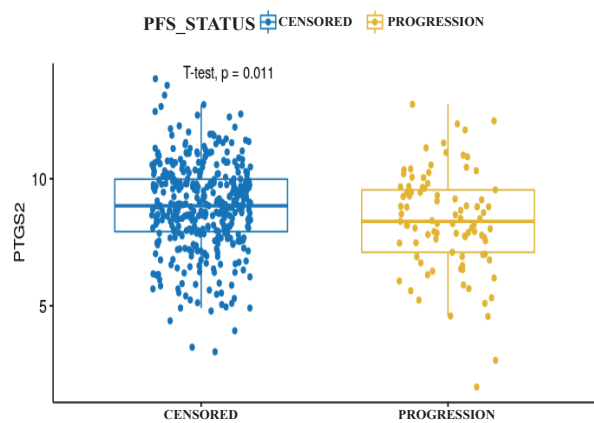

K

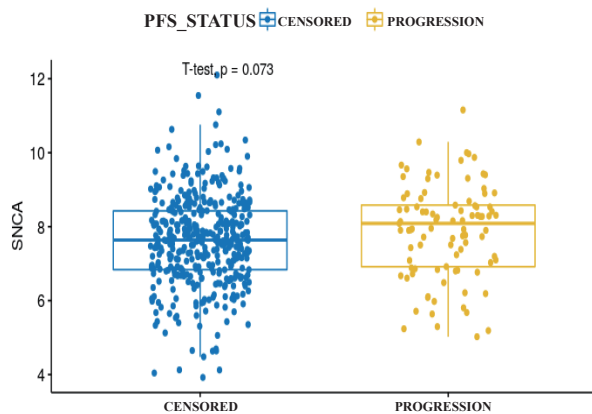

Supplement: Supplementary Materials — Supplementary forms and pictures are in supplementary files. [file 3758219.f1.zip › Figure.S5.pdf]
